# Correction: Al Madhoun et al. Randomized Clinical Trial: Bone Bioactive Liquid Improves Implant Stability and Osseointegration. *J. Funct. Biomater.* 2024, *15*, 293

**DOI:** 10.3390/jfb16050171

**Published:** 2025-05-09

**Authors:** Ashraf Al Madhoun, Khaled Meshal, Neus Carrió, Eduard Ferrés-Amat, Elvira Ferrés-Amat, Miguel Barajas, Ana Leticia Jiménez-Escobar, Areej Said Al-Madhoun, Alaa Saber, Yazan Abou Alsamen, Carles Marti, Maher Atari

**Affiliations:** 1 Department of Animal and Imaging Core Facilities, Dasman Diabetes Institute, Dasman 15462, Kuwait; ashraf.madhoun@dasmaninstitute.org; 2 Biointelligent Technology Systems SL, C/Diputaccion 316, 3D, 08009 Barcelona, Spain; mplant21@yahoo.com (K.M.); eeduard.fa@institutferresamat.com (E.F.-A.); miguel.barajas@unavarra.es (M.B.); areejalma222@gmail.com (A.S.A.-M.); asaber@biointelligentsl.com (A.S.); dryazanabualsamen@gmail.com (Y.A.A.); martipages.c@gmail.com (C.M.); 3 Periodontology Department, Universitat Internacional de Catalunya (UIC), C/Josep Trueta s/n, 08195 Barcelona, Spain; neuscarriober@gmail.com; 4 Oral and Maxillofacial Surgery Department, Universitat Internacional de Catalunya (UIC), St Josep Trueta s/n, 08195 Barcelona, Spain; 5 Oral and Maxillofacial Surgery and Pediatric Dentistry Department, Universitat Internacional de Catalunya (UIC), St Josep Trueta s/n, 08195 Barcelona, Spain; eferresamat@uic.es; 6 Biochemistry and Molecular Biology Department, Universidad Pública de Navarra, 31006 Pamplona, Spain; 7 Inves Biofarm, Avd. Conocimiento, 34, 18016 Granada, Spain; anajimenez@invesbiofarm.com; 8 Oral and Maxillofacial Surgery Department, Hospital Clinic de Barcelona, 08036 Barcelona, Spain

Error in Figure and Figure Legend

In the original publication [1], there was a mistake in Figure 3 and its legend. The manufacturer of the implant should be “MIS” rather than “Galaxy”. The correct figure and legend appear below. 

Text Correction

There was an error in the original publication. The manufacturer of the implant should be “MIS” rather than “Galaxy” in Sections 3.4 and 4.

A correction has been made to Section 3.4:

The surface characterization of MIS TS and MIS TSA discs was performed using SEM and AFM. SEM images of the MIS TS disc surface at various magnifications revealed a consistent “uniform-rough” texture (Figure 3A). The SEM analysis revealed that MIS surfaces exhibit a distinct topographical pattern characterized by a combination of macro-textured roughness due to sandblasting with large-grit particles and micro-textured features resulting from acid etching. Sandblasting imparts a rough, uneven texture with noticeable grooves and ridges, while the subsequent acid etching process creates a porous, micro-rough surface.

AFM measurements were employed to evaluate the surface morphology of MIS TS and MIS TSA. The 3D topography images of MIS TS showed considerably higher roughness, measuring approximately 100 nm (Figure 3(Ba)), in comparison with the BBL-treated surface disc, which exhibited a roughness of about 33 nm (Figure 3(Ca)). These differences in the surface characteristics are further illustrated in the 2D images, where variations in the color depth levels are evident (Figure 3(Bb,Cb)). Notably, the BBL treatment creates a liquid environment that reduces the disc roughness and stiffness, as visualized in the AFM mechanical mapping (Figure 3(Cc)), in comparison to untreated MIS TS discs (Figure 3(Bc)).

A correction has been made to Section 4:

4. Discussion 

The findings of this study shed light on the interaction between the BBL and Galaxy TS surfaces and MIS TS, contributing to ongoing efforts to enhance the bioreactivity of SLA (sandblasted, large-grit, acid-etched) surfaces, known for their favorable roughness characteristics for implantation [37]. While SLA surfaces have demonstrated good osseointegration properties, further improvement through surface modifications using bioactive materials has been explored extensively [38–40].

The authors state that the scientific conclusions are unaffected. This correction was approved by the Academic Editor. The original publication has also been updated.

## Figures and Tables

**Figure 3:**
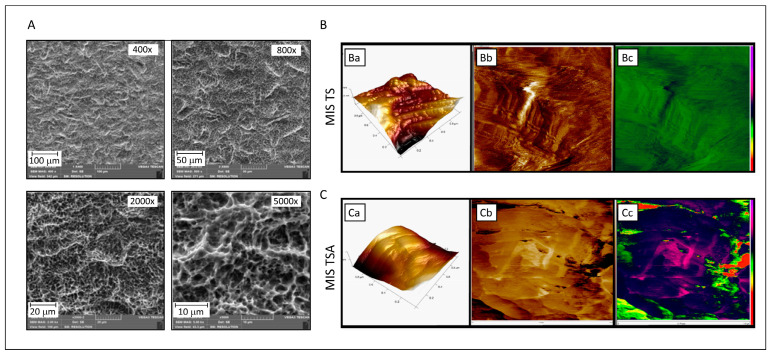
Characterization of titanium alloy discs of the MIS implant surface. (**A**) SEM analysis of MIS TS implant at different magnifications (400×, 800×, 2000×, and 5000×) after alumina blasting and acid etching. (**B**,**C**) AFM analysis of discs of MIS TS and MIS TSA surfaces, respectively. (**Ba**) MIS TS surface: 1 × 1 micron 3D topographic image (surface roughness Rq parameter is 1.3 nm) showing homogeneity on the sample surface. (**Bb**,**Bc**) MIS TS surface: 1 × 1 micron 3D topographic image (surface roughness is 1.3 nm), where no substrate structure can be observed on the surface. (**Ca**) MIS TSA surface: 1 × 1 micron 3D topographic image (surface roughness Rq parameter is 1.3 nm) and 1 × 1 micron phase images (in two different color scales) corresponding to the previous topographic image, showing homogeneity on the sample surface. (**Cb**,**Cc**) MIS TS surface: 1 × 1 micron 3D topographic image (surface roughness is 74.5 nm) and 1 × 1 micron phase images corresponding to the previous topographic image. The surface appears less rough than in the blank sample, implying that the substrate structure is coated with BBL, which reduces the smoothness.
